# Molecular Diagnosis and Novel Therapies for Neuromuscular Diseases

**DOI:** 10.3390/jpm10030129

**Published:** 2020-09-16

**Authors:** Rika Maruyama, Toshifumi Yokota

**Affiliations:** 1 Department of Medical Genetics, Faculty of Medicine and Dentistry, University of Alberta, Edmonton, AB T6G 2H7, Canada; yokotama@ualberta.ca; 2 The Friends of Garrett Cumming Research & Muscular Dystrophy Canada, HM Toupin Neurological Science Research Chair, Edmonton, AB T6G 2H7, Canada

**Keywords:** Duchenne/Becker muscular dystrophy (DMD/BMD), amyotrophic lateral sclerosis (ALS), viltolarsen, eteplirsen, golodirsen, phosphorodiamidate morpholino oligomers (PMOs), exon skipping, CRISPR (clustered regularly interspaced short palindromic repeat)/Cas9 (CRISPR associated protein 9)-mediated genome editing, multiplex ligation amplification (MLPA), next-generation sequencing (NGS)

## Abstract

With the development of novel targeted therapies, including exon skipping/inclusion and gene replacement therapy, the field of neuromuscular diseases has drastically changed in the last several years. Until 2016, there had been no FDA-approved drugs to treat Duchenne muscular dystrophy (DMD), the most common muscular dystrophy. However, several new personalized therapies, including antisense oligonucleotides eteplirsen for DMD exon 51 skipping and golodirsen and viltolarsen for DMD exon 53 skipping, have been approved in the last 4 years. We are witnessing the start of a therapeutic revolution in neuromuscular diseases. However, the studies also made clear that these therapies are still far from a cure. Personalized genetic medicine for neuromuscular diseases faces several key challenges, including the difficulty of obtaining appropriate cell and animal models and limited its applicability. This Special Issue “Molecular Diagnosis and Novel Therapies for Neuromuscular/Musculoskeletal Diseases” highlights key areas of research progress that improve our understanding and the therapeutic outcomes of neuromuscular diseases in the personalized medicine era.

Neuromuscular diseases include a large number of different medical conditions that affect the peripheral nervous system and muscle [1,2]. Many of them are incurable genetic diseases [3,4]. In the last few decades, numerous genes have been identified that directly or indirectly affect neuromuscular function [5]. Subsequently, studies on various cell and animal models have substantially contributed to our knowledge of the molecular mechanisms underlying neuromuscular diseases and therapeutics [6,7,8,9,10]. These studies directly led to the development of the currently available personalized genetic medicine, including antisense oligonucleotide-mediated exon skipping therapies [11,12,13,14].

A key challenge in genetic diseases, however, is the difficulty of obtaining cell and animal models that faithfully recapitulate the disease phenotype [15]. In addition, many animal models are often not very useful in testing mutation-specific therapies including exon skipping and genome editing because of the differences in the mutation patterns and gene sequences between humans and animal models [16]. Newly developed models, including humanized models and clustered regularly interspaced short palindromic repeat (CRISPR)-generated animal models, effectively addressed these challenges. A couple of review articles written by Lim et al., one of which is included in this Special Issue, discuss this challenge and future perspectives [15,17].

Another key area in the personalized medicine era is an accurate and cost-effective genetic diagnosis [18]. In this Special Issue, Nakamura reviews the recent progress of accurate diagnosis methods and therapeutic strategies for Duchenne muscular dystrophy (DMD), the most common lethal muscle disease [19]. Recent advances in genetic diagnosis, such as multiplex ligation amplification (MLPA) and next-generation sequencing (NGS), have greatly enhanced our ability to pinpoint mutations. In addition to the accurate genetic diagnosis, the characterization of mutations including genotype-phenotype correlation studies of exon skip-equivalent in-frame mutations is becoming increasingly important in order to optimize the effects of exon skipping therapies. For example, as Echigoya et al. pointed out in their article, exons 45–55 skipping and exons 3–9 skipping may lead to a milder phenotype, as seen in milder Becker muscular dystrophy (BMD) patients, compared to smaller in-frame deletions, which are more often associated with DMD [20].

There are several approaches to mutation-specific personalized genetic therapy for DMD. These approaches aim to restore dystrophin expression using different techniques, including stop-codon read-through, antisense oligonucleotide-mediated exon skipping, and genome editing. In this Special Issue, the former two approaches are discussed in detail by Shimizu-Motohashi et al. [21]. Genome-editing therapy is still in its infancy, facing many challenges, but it has already demonstrated promising effects in cell and animal models [22]. In this Special Issue, Lim et al. discuss the promises and challenges of this approach [17].

Although significant progress has been made in DMD and spinal muscular atrophy (SMA) therapeutics, patients with most neuromuscular diseases, such as amyotrophic lateral sclerosis (ALS), still have no effective targeted treatment option available [23]. Since many genes and mechanisms are involved in ALS, it is clearly a more challenging therapeutic target of personalized medicine. In this Special Issue, Morgan et al. discuss the recent developments of personalized medicine and molecular interaction networks in ALS [24]. 

In conclusion, we welcome a new era of personalized genetic medicine as we move forward enthusiastically towards the next generation of therapeutic technologies. We hope this collection of articles can provide readers with a useful introduction to molecular diagnosis and novel therapies for neuromuscular diseases in the personalized medicine era.
